# Supplementary material for: Identification of photoperiod-induced specific miRNAs in the adrenal glands of Sunite sheep (Ovis aries)
Source: Front Vet Sci. 2022 Jul 22;9:888207. doi: 10.3389/fvets.2022.888207 (PMC9354845; doi:10.3389/fvets.2022.888207)
Supplement: Supplementary file 1 [file Data_Sheet_1.ZIP › Supplementary file/miRDeep2 file.zip!/miRDeep2 file/Novel_Structure/SP42A1/Novel_249.pdf]

Provisional ID	:	NC_019463.2_9759
Score total	:	2.3
Score for star read(s)	:	-1.3
Score for read counts	:	0
Score for mfe	:	2
Score for randfold	:	1.6
Score for cons. seed	:	
Total read count	:	291
Mature read count	:	291
Loop read count	:	0
Star read count	:	0

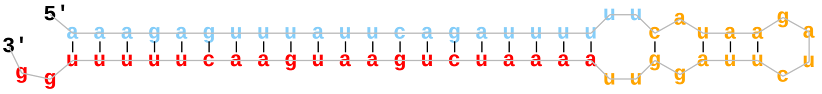

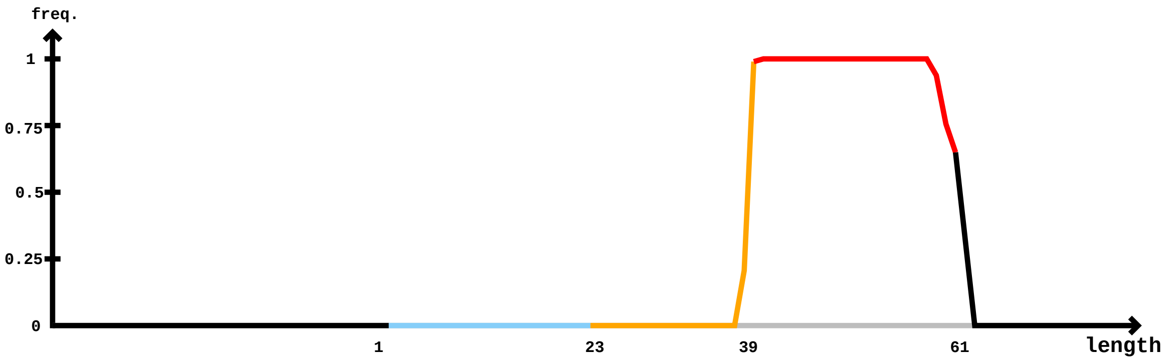


```
                                    Star                                Mature
5'- uacuauuaacucuaaauaguuaauagguuggccaaagaguuuauucagauuuuuucauaagaucuuagguuaaaaucugaaugaacuuuuuggccaaguuacuaauuuaau   -3'   exp
    ..(((((((((......))))))))).((((((((((((((((((((((((((..(.(((....))).)..))))))))))))))))))))))))))..............   reads   mm   sample
    ......................................................................Aaaaaucugaaugaacuuuu.....................   17      1    seq
    ......................................................................uaaaaucugaaugaacuuuuu....................   1       0    seq
    ......................................................................Aaaaaucugaaugaacuuuuu....................   42      1    seq
    .......................................................................aaaCucugaaugaacuuuu.....................   1       1    seq
    .......................................................................aaaGucugaaugaacuuuuu....................   1       1    seq
    .......................................................................aaaaucugaaugaacuuuuA....................   5       1    seq
    .......................................................................aaaaAcugaaugaacuuuuu....................   2       1    seq
    .......................................................................aaaaucugaaugaacuuuuug...................   30      0    seq
    .......................................................................aaaaucugaaugaacuuuuuC...................   1       1    seq
    .......................................................................aaaCucugaaugaacuuuuugg..................   4       1    seq
    .......................................................................aaaaucugaaugaacuuuuuUg..................   2       1    seq
    .......................................................................aaaaucugaaugaacuuuuugA..................   52      1    seq
    .......................................................................aaaaucugaaugaacuuuuugg..................   124     0    seq
    .......................................................................aaaaucugaaugaacuuuuuggU.................   6       1    seq
    ........................................................................aaaucugaaugaacuuuuu....................   1       0    seq
    ........................................................................aaaucuAaaugaacuuuuu....................   1       1    seq
    ........................................................................aaaucugaauAaacuuuuuggc.................   1       1    seq
```
